# Rapid detection and curation of conserved DNA via *enhanced*-BLAT and *EvoPrinterHD *analysis

**DOI:** 10.1186/1471-2164-9-106

**Published:** 2008-02-28

**Authors:** Amarendra S Yavatkar, Yong Lin, Jermaine Ross, Yang Fann, Thomas Brody, Ward F Odenwald

**Affiliations:** 1 Division of Intramural Research, Information Technology Program, NINDS, NIH, Bethesda, Maryland, USA; 2 The Neural Cell-Fate Determinants Section, NINDS, NIH, Bethesda, Maryland, USA

## Abstract

**Background:**

Multi-genome comparative analysis has yielded important insights into the molecular details of gene regulation. We have developed *EvoPrinter*, a web-accessed genomics tool that provides a single uninterrupted view of conserved sequences as they appear in a species of interest. An *EvoPrint *reveals with near base-pair resolution those sequences that are essential for gene function.

**Results:**

We describe here *EvoPrinterHD*, a 2^nd^-generation comparative genomics tool that automatically generates from a single input sequence an enhanced view of sequence conservation between evolutionarily distant species. Currently available for 5 nematode, 3 mosquito, 12 *Drosophila*, 20 vertebrate, 17 *Staphylococcus *and 20 enteric bacteria genomes, *EvoPrinterHD *employs a modified BLAT algorithm [*enhanced*-BLAT (*e*BLAT)], which detects up to 75% more conserved bases than identified by the BLAT alignments used in the earlier *EvoPrinter *program. The new program also identifies conserved sequences within rearranged DNA, highlights repetitive DNA, and detects sequencing gaps. *EvoPrinterHD *currently holds over 112 billion bp of indexed genomes in memory and has the flexibility of selecting a subset of genomes for analysis. An *EvoDifferences *profile is also generated to portray conserved sequences that are uniquely lost in any one of the orthologs. Finally, *EvoPrinterHD *incorporates options that allow for (1) re-initiation of the analysis using a different genome's aligning region as the reference DNA to detect species-specific changes in less-conserved regions, (2) rapid extraction and curation of conserved sequences, and (3) for bacteria, identifies unique or uniquely shared sequences present in subsets of genomes.

**Conclusion:**

*EvoPrinterHD *is a fast, high-resolution comparative genomics tool that automatically generates an uninterrupted species-centric view of sequence conservation and enables the discovery of conserved sequences within rearranged DNA. When combined with *cis*-Decoder, a program that discovers sequence elements shared among tissue specific enhancers, *EvoPrinterHD *facilitates the analysis of conserved sequences that are essential for coordinate gene regulation.

## Background

Comparative analysis of orthologous DNA has revealed that many *cis*-regulatory enhancers contain multi-species conserved sequences (MCSs) that are essential for their transcriptional regulation (reviewed by [1-4]). We have previously described *EvoPrinter *and *cis*-Decoder, both web-accessed tools for discovering and comparing conserved sequences that are shared among three or more orthologs [4,5]. Generated from superimposition of multiple pair-wise BLAT alignments [6], an *EvoPrint *provides an ordered uninterrupted representation of conserved sequences as they exist in the genome of interest. When multiple species are included in the analysis, near base-pair resolution of conserved sequences required for gene function can be achieved. For example, when 12 *Drosophila *species, representing ~200 million years of cumulative evolutionary divergence, are included in the *EvoPrint *process, one can identify sequences that are essential for *cis*-regulatory function (both enhancers and minimal promoters), conserved protein encoding sequences, and micro-RNA binding sites. *EvoPrinterHD *is a second-generation alignment tool that automates the comparative analysis to rapidly identify a significantly higher percentage of conserved sequences shared among evolutionarily distant orthologs even if they exist within rearranged DNA. In contrast to most comparative multi-sequence alignment tools (reviewed by [7]), which display columns of sequences that contain gaps to optimize alignments, the species-centric *EvoPrint *is a single uninterrupted sequence and thus displays more bases in a single view than is possible with conventional alignments. In addition, the uninterrupted readout allows for the rapid extraction and automated curation of conserved DNA from the genome of interest.

At the core of the original multi-genome *EvoPrinter *alignment algorithms is the BLAT algorithm [6] for pairwise alignments. Although BLAT alignments generate uninterrupted representations of the aligning regions, one drawback of BLAT when performing alignments of evolutionarily distant DNAs, as initially noted by Kent [6], is that short regions of homology that span the non-overlapping 11-mers go undetected. We developed *e*BLAT to overcome the inability of BLAT to detect these short blocks of homology. To accomplish this, each genome is indexed three independent ways, each staggered differently; additionally, the alignment parameters have been adjusted to enhance the detection of short blocks of sequence conservation. By performing three independent alignments using the staggered indices with the optimized alignment parameters and then superimposing the resulting alignments to show all aligning sequences, the overall detection of conserved sequences has been improved by as much as 75% when evolutionary distant orthologous sequences are aligned.

In addition to the automated alignments for bacteria, nematode, mosquito, *Drosophila*, and vertebrate genomes, and the higher *e*BLAT resolution, *EvoPrinterHD *includes algorithms that search the intra-genomic aligning regions for rearrangements, duplications and sequencing gaps. *EvoPrints *generated with composite *e*BLATs highlight conserved sequences within the reference DNA irrespective of genomic rearrangements within one or more of the aligning regions. Four additional programs have been added: (1) an *EvoDifferences *profile, portraying in a single view the conserved sequences that are detected in all but one of the species included in the *EvoPrint*; (2) input reference DNA exchange, allowing for detection of species-specific changes in the less-conserved DNA flanking MCSs; (3) automated extraction and curation of conserved sequence blocks (CSBs), facilitating their comparative analysis [4], and (4) for bacteria, an *EvoUnique *print that highlights unique or uniquely shared sequences among subsets of genomes. Due in part to its speed and flexibility of genome selection, *EvoPrinterHD *interfaces well with other web-accessed tools. The time required to undertake a comparative genome analysis of sequences that contain putative *cis*-regulatory enhancers is significantly reduced. For example, a 12 *Drosophila EvoPrint *analysis and curation of CSBs within a 2 Kb genomic region that contains a cluster of transcription factor DNA-binding sites (discovered using the *FlyEnhancer *genome motif search tool [8]) requires less than 30 seconds. Once CSBs are discovered, subsequent analysis via *cis*-Decoder algorithms enable the generation of conserved sequence tag libraries that further facilitate enhancer comparative studies.

## Results and Discussion

The following is a description of the sequential steps and accompanying algorithms used by *EvoPrinterHD *to identify conserved sequences shared among multiple genomes. Instructions and a tutorial for optimizing its use can be accessed at the *EvoPrinterHD *web site [9].

### Genome Indexing

In addition to the original non-overlapping 11-mer genomic index of BLAT [6], *EvoPrinterHD *indexes each genome into a second set of non-overlapping 11-mers, offset by four base pairs from the initial indexing, and into a third set of non-overlapping 9-mers. The resulting staggered indexing increases the likelihood that homologous regions missed by any one of the individual indices will be identified. The use of multiple genome indices and optimization of the alignment phase parameters (see below) is the basis of the enhanced detection of conserved sequences between evolutionarily distant orthologous DNAs.

*EvoPrinterHD *currently holds in memory three independent indices of each of 37 bacteria, 3 mosquito, 5 nematode, 12 *Drosophila *and 20 vertebrate genomes, representing ~112 billion bp in total memory.

### Modification of BLAT search and alignment parameters

The alignment sensitivity of *EvoPrinterHD *for the discovering short blocks of conserved sequence homology between evolutionary distant orthologs was increased by optimizing the Genomic Finding (gf) client program parameters of the original BLAT algorithm [6]. The search and alignment parameters were adjusted by: (1) optimizing the stringency factor for low homology alignments by increasing it from 0.0005 to 0.001, (2) reducing the initial expansion gap between adjacent hits from a setting of four to three, (3) reducing the additional expansion gap penalty from three to one, (4) maximizing the allowable gaps and inserts from 12 to 16, and (5) changing the value of allowable codon gap parameter from two to three to optimize for codon polymorphisms in open reading frames.

### Detecting conserved sequences with EvoPrinterHD algorithms

To maximize the identification of short CSBs between evolutionary divergent orthologs, *EvoPrinterHD *generates 3 different input reference DNA vs. test genome BLAT alignments to the same aligning region using the three indices described above. As an output of the client program, *EvoPrinterHD *then generates a superimposed composite of the 3 different alignments. The algorithm does this by first creating an array of nucleotide strings of each of the 3 input reference DNA BLAT alignment sequences and then loops through the strings one base at a time, outputting a capital letter when at least one of the 3 readouts has an aligning base at that position, thereby generating a composite readout that displays all conserved bases. The program also generates BLAT readouts of the test genome aligning region and both are stored in memory for later analysis, *EvoPrint *generation and for exchange of input reference DNA, accomplished by selecting one of the aligning region sequences as the new reference sequence to reinitiate the analysis. The algorithm also generates *e*BLATs for the second and third highest score aligning regions for each of the selected genomes.

The mosquito, nematode, *Drosophila *and *Staphylococcus EvoPrinterHD *algorithms automatically generate, respectively, 27, 45, 108 and 153 pairwise BLAT alignments, assembles 9, 15, 36, and 51 *e*BLAT readouts, and then superimposes the individual pairwise *e*BLAT alignments (3 per genome) to generate a color-coded composite-*e*BLAT (c*e*BLAT) for each aligning region. The vertebrate *EvoPrinterHD *and enteric bacteria *EvoPrinterHD *both generate up to 180 pairwise BLAT alignments assembling 60 *e*BLAT readouts and 20 c*e*BLATs. To reduce alignment times, *EvoPrinterHD *algorithms currently employ two *Dell PowerEdge *(2.8 GHz/64 GB RAM; 6950 series) dual quad-core processor servers operating in parallel with the *RedHat Enterprise Linux *5 operating system and the Network File System to simultaneously query multiple indexed genomes.

**Figure 1:**
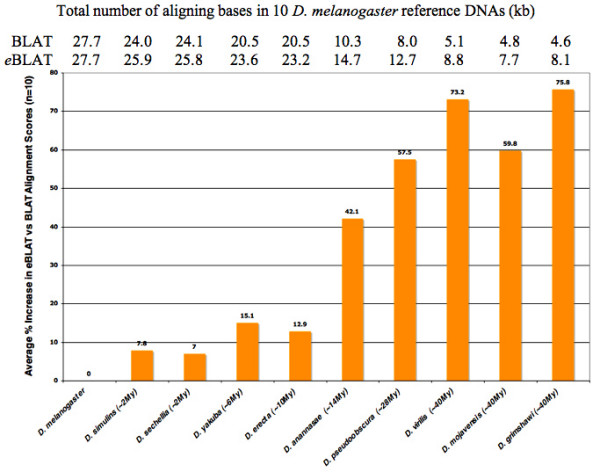
**Increased identification of conserved DNA in evolutionary distant orthologs via *enhanced*-BLAT pairwise alignments**. Shown are the total number of aligning bases in pairwise BLAT and pairwise *enhanced*-BLAT alignments from 10 different *Drosophila melanogaster *genomic regions that contain conserved sequence blocks (1.3 to 4.7 kb; 27.7 kb in total) aligned to the orthologous DNAs from *D. melanogaster*, *D. simulans*, *D. sechellia*, *D. yakuba*, *D. erecta*, *D. ananassae*, *D. pseudoobscura*, *D. virilis*, *D. mojavensis *or *D. grimshawi*. The average percent increase in the number of *e*BLAT aligning bases vs. BLAT alignments is also shown. The approximate evolutionary separation/divergence time (in million years) between *D. melanogaster *and the other Drosophilids is indicated in brackets.

To assess the efficacy of *e*BLAT alignments in comparison to the original BLAT, we compared the pairwise alignment scores (the total number of aligning bases in the input DNA) of *e*BLAT to those obtained with BLAT, using 10 different intergenic regions from the *Drosophila melanogaster *genome (Figure 1). The genomic fragments (1.3 to 4.7 kb in length -totaling 27.7 kb) were selected because they each had been previously shown to contain *cis*-regulatory transcriptional enhancers. They include DNA flanking the following genes: *gooseberry-neuro *[10], *snail *[11], *hunchback *[12], *slit *(enhancer 2.6 RV) [13], *string *(enhancer 5.8) [14], *atonal *[15], *Sex combs reduced *(enhancer 3.0 RR) [16], *Toll *(enhancer 6.5 RL/LR) [13] and *Par domain protein 1 *(1^st ^intron enhancer) [17]. Nine of these regions are described in *RedFly*, the regulatory element database for *Drosophila *[18], while the tenth, the *nerfin-1 *neuroblast enhancer was identified by A. Kuzin in the Odenwald laboratory (personal communication). In addition, twelve genome *EvoPrint *analysis of each of the ten intragenic regions revealed that each region contained highly conserved sequences that were shared by all Drosophilids (data not shown). As demonstrated in Figure 1, the pairwise *e*BLAT alignment exhibited only a modest increase in the identification of shared sequences between closely related species over the conventional BLAT alignment; however, *e*BLAT identified significantly more conserved sequences when the *D. melanogaster *genomic fragments were aligned to the more evolutionarily distant orthologs. The increased identification of shared sequences varied from a 7.5% increase for *D. simulans *(evolutionary divergent time from *D. melanogaster *is ~2 My) to 74.8% for *D. grimshawi *(separated from *D. melanogaster *for ~40 My). The same enhanced discovery of sequence conservation was also observed when evolutionarily distant nematode or vertebrate species were compared. For example, *e*BLAT alignments between *C. elegans *and *C. briggsae *or human and *Xenopus *orthologous DNAs both identified greater than 70% more shared sequences when compared to original BLAT alignments (data not shown).

**Figure 2:**
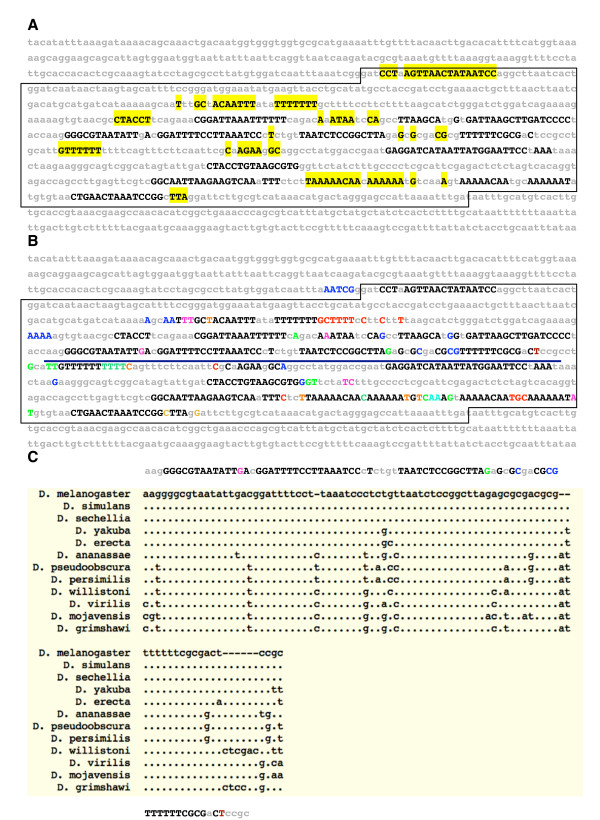
***EvoPrints *generated with *e*BLAT alignments reveal additional conserved sequences when compared to the original method**. A) Shown is a composite *EvoPrint *of the *Drosophila melanogaster Krüppel *central domain (CD2) enhancer region generated by superimposing an *EvoPrint *generated from *e*BLAT alignments and a second prepared from BLAT alignments. Pairwise alignments between *D. melanogaster *and *D. sechellia, D. simulans, D. erecta, D. yakuba, D. ananassae, D. pseudoobscura, D. persimilis, D. virilis, D. willistoni, D. mojavensis and D. grimshawi *were used to generate both *EvoPrints*. Conserved sequences identified by both procedures are shown as uppercase black nucleotides and yellow highlighted nucleotides represent the additional sequences recognized by *EvoPrinterHD*. The boxed region contains the *cis*-regulatory DNA required for enhancer function as determined by Hoch et al. [9]. B) An *EvoDifferences *profile identifies those DNA sequences that are shared by all but one of the species included in the analysis. As in the *EvoPrint*, black uppercase letters indicate sequences shared by all species and colored uppercase letters, which denote individual species, represent sequences that were not detected by the *e*BLAT alignment for just one of the genomes included in the *EvoPrint *analysis (*D. erecta*, dark-red; *D. yakuba*, teal; *D. pseudoobscura*, light-blue; *D. persimilis*, brown; *D. ananassae*, pink; *D. virilis*, orange; *D. willistoni*, blue; *D. mojavensis*, green; *or D. grimshawi*, red). The underline indicates the region of the *EvoDifferences *profile that is compared with the alignments obtained from the UCSC genome browser (shown in panel C). C) Comparison of the *EvoDifferences *profile with the UCSC genome alignments. Shown is the underlined sequence in panel (B) aligned to the corresponding alignments obtained at the *Drosophila *UCSC comparative genome bioinformatics web site.

Another measure of *e*BLAT efficacy in identifying evolutionary conservation is to compare the detection of conserved sequences when *e*BLAT vs. BLAT alignments are used to generate an *EvoPrint*. To demonstrate the increased alignment sensitivity of *e*BLAT over BLAT in the *EvoPrint *analysis, the *Drosophila melanogaster Krüppel *central domain enhancer [19] was *EvoPrinted *using 11 of the *Drosophila *species (Figure 2A). The original *EvoPrinter *(which uses the BLAT algorithm) detected a total of 169 conserved bases compared with 254 conserved bases identified with an *e*BLAT generated EvoPrint – a 50% increase in alignment recognition. In addition, the *EvoDifferences *profile identified additional bases (shown in color) that are conserved in all but one of the genomes used to generate the *EvoPrint *(Figure 2B and see below).

We also compared *EvoPrinterHD*-generated *EvoPrints *to multi-genome alignments obtained from the UCSC comparative genome bioinformatics alignment program [20,21]. The alignment resolution of *EvoPrinterHD *is equivalent to the multi-species UCSC alignments in detecting CSBs. The two alignment programs detect the same conserved sequences with 93% to 95% correspondence in five different enhancers compared (Figure 2C; and data not shown).

### EvoPrinterHD repeat finder

**Figure 3:**
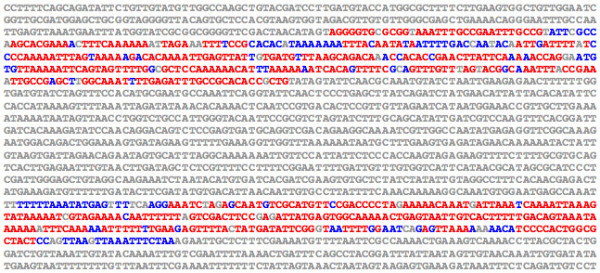
***EvoPrinterHD *repeat finder algorithm identifies repetitive elements within the input DNA**. The repeat finder algorithm superimposes the three highest scoring *e*BLAT input reference DNA to reference genome alignments to reveal those sequences within the input DNA that are repeated within the input DNA itself and/or elsewhere in the reference genome. Single-copy repeat sequences, identified just once in the second or third highest scoring *e*BLATs but not in both, are highlighted by blue-colored bases. Multiple (≥ 3 copies) repeats are highlighted with red-colored bases. Shown is a 1,958 bp genomic fragment that flanks the 3' end of the *Caenorhabditis elegans egl-26 *gene (+5,290 to +7,248 bp from the start of transcription) that was initially part of a 20 kb input DNA repeat finder readout. Note, the single copy repeat (blue-colored) sequences that flank the multi-copy repeat sequences (red-colored) indicate that one of the repeat copies located elsewhere in the reference genome is more homologous to the input DNA repeat sequence than with its other repeat family members.

One prominent feature of all bacteria and metazoan genomes is that they harbor diverse populations of repetitive elements that range in copy number from single duplications to thousands of transposable elements dispersed throughout the genome. Given that many of these repeats contain highly conserved sequences that may interfere with alignments between evolutionary distant orthologs, it is important to first identify the repetitive sequence(s) within the reference genome before comparative analysis is considered. To accomplish this, the *EvoPrinterHD *repeat finder algorithm superimposes the first, second and third highest scoring *e*BLAT alignments of the input DNA to its own genome and then color-codes the readout to identify single or multiple repeat sequences within the input reference DNA (Figure 3). Sequences that have one additional copy in the reference genome are noted with blue-colored uppercase bases while those that are present three or more times are highlighted with red-colored bases. The algorithm also reveals if one of the multiple repeat sequences is more homologous to the repeat present in the input DNA by highlighting single repeat sequences that flank the core multi-repeat element (Figure 3). By underlining repeat sequences in the *EvoPrint *and *EvoDifference *readouts potential 'false positive' alignments that have their origin in repetitive elements are highlighted.

### Alignment scorecard

**Figure 4:**
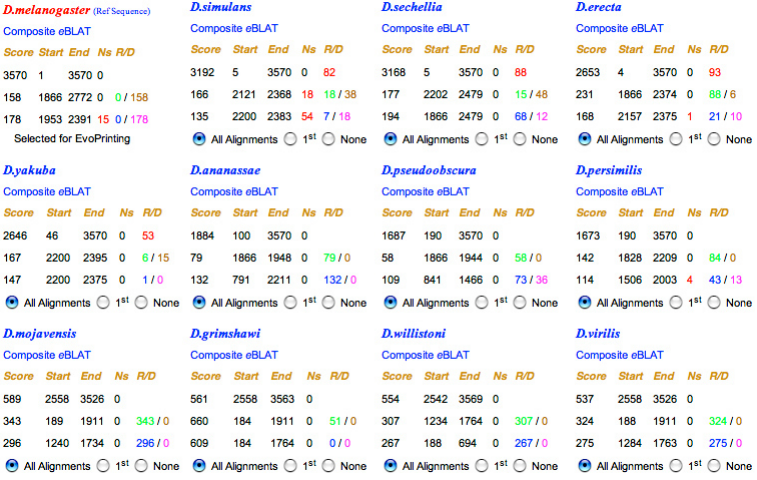
***EvoPrinterHD *alignment scorecard**. A) Once the *e*BLAT alignment phase is completed, the algorithm initially displays the data in a tabular/scorecard form. The total number of aligning bases for each pair-wise alignment (the homology score) is shown along with the position of the first and last aligning bases within the input reference DNA sequence. The genomes are arrayed in descending order of alignment score and the 3 highest pairwise alignment scores for each species are shown. The intra-genomic algorithm compares the second and third scoring alignments of each genome to its highest scoring alignment to identify potential regions that harbor conserved sequences that have either rearranged and/or duplicated, in addition to identifying sequencing gaps within the aligning regions. The input reference DNA *e*BLAT readouts and the aligning region BLAT for each alignment can be accessed by clicking on the species name and links to the Composite eBLATs are also provided. Each species can be selected or deselected for *EvoPrinting *and by default, *EvoPrinterHD *selects the 6 highest scoring species for generating the initial *EvoPrint *and *EvoDifferences *profile readouts. "Ns" represent the number of sequencing gaps detected in each of the aligning regions. The "R" value (indicative of a putative rearrangement) for the second and third alignments indicates the number of aligning bases not detected in the first alignment and the "D" value (indicative of a putative duplication) is the number of aligning bases shared with the first alignment. A link in provided for changing the input reference DNA to the aligning region of one of the other species. Shown is the alignment scorecard for a 3,570 bp *Drosophila melanogaster *sequence that is located 6 kb upstream of the *fushi tarazu *gene. As indicated by the "R/D" values for each of the species, the intra-genomic comparative program has identified potential rearrangements and duplications. The color code reveals 1) whether the R or D value is derived from the second or third alignment and 2) whether a putative rearrangement or duplication has been detected.

As a prelude to generating an *EvoPrint*, the inter-genome comparative program first displays the results of the different alignments in a tabular form referred to here as the alignment scorecard (Figure 4 and see examples at the website tutorial [9]). The scorecard shown in Figure 4 was generated from a *cis*-regulatory enhancer region associated with the *Drosophila melanogaster fushi tarazu *gene (see below for more details). The alignment score for each species' *e*BLAT alignments shows the total number of aligning bases in the input reference DNA. The positions of the first and last aligning bases in the input reference DNA are also noted, along with the number of sequencing gaps detected in the aligning regions of the test genomes and the total number of "Ns" (the presumed number of missing bases as indicated in the database). Links to the alignment readouts for each species are provided on the scorecard, allowing the user to view the individual reference DNA and test species alignments. A second link for each species leads to a color-coded composite *e*BLAT of all 3 of its alignments that highlights sequence rearrangements and/or duplications in the test species (see below). The data is arrayed in a descending order of alignment scores. By default, top scoring genomes with no sequencing gaps in their highest scoring alignments are selected for the initial *EvoPrint *analysis. After the initial *EvoPrint *and *EvoDifferences *profile is examined, it is recommended that the lower scoring species be included one at a time to extend the evolutionary comparison (see below).

### Identification of rearranged and duplicated conserved sequences

**Figure 5:**
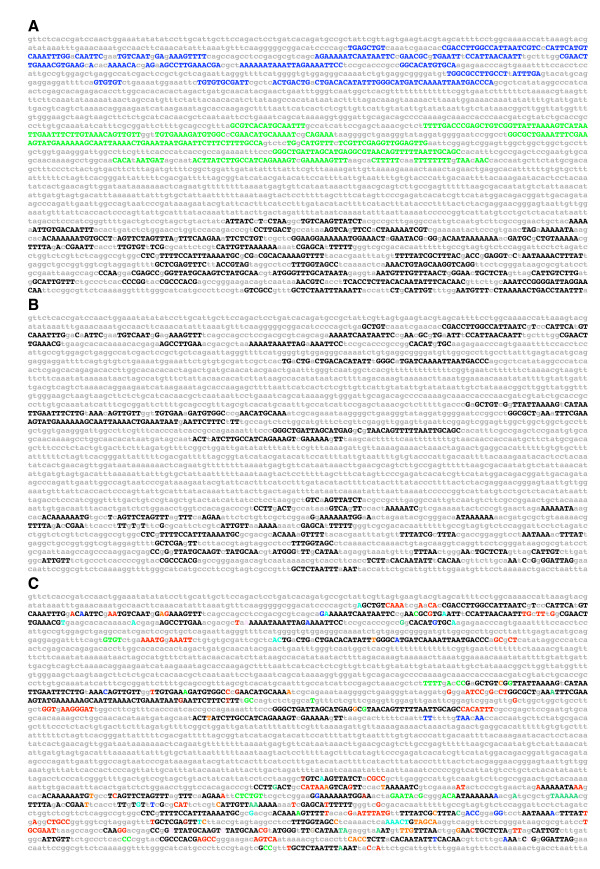
**Intra-species c*e*BLATs and composite-*EvoPrints *identify conserved sequences within the input reference DNA that have rearranged in the aligning regions of other genomes**. A) Shown is a *D. melanogaster *(reference DNA) to *D. virilis *c*e*BLAT alignment that spans a 3,570 bp sequence located upstream of the *fushi tarazu *gene (-7184 to -3,434 bp from its transcription start). Black-colored uppercase nucleotides represent aligning bases found only in the highest scoring *D. virilis e*BLAT alignment, green-colored bases identify aligning bases that are unique to the second highest scoring alignment and blue-colored bases are aligning bases unique to the third highest score *e*BLAT alignment. B) Shown is an *EvoPrint *of the input *D. melanogaster *sequence shown in (A) that was generated with c*e*BLATs of the *D. simulans*, *D. sechellia*, *D. yakuba*, *D. erecta*, *D. ananassae*, *D. pseudoobscura*, *D. persimilis*, *D. virilis*, *D. mojavensis*, *D. grimshawi *and *D. willistoni *alignments. C) The accompanying *EvoDifferences *profile of the *EvoPrint *shown in (B). Black uppercase letters are aligning bases shared by all species examined. Colored uppercase letters, which denote individual species, represent sequences that were not aligned in the c*e*BLAT for just one of the genomes included in the analysis (*D. simulans*, teal; *D. sechellia*, dark-red; *D. yakuba*, brown; *D. erecta*, light-blue; *D. ananassae*, orange; *D. pseudoobscura*, pink; *D. virilis*, blue; *D. mojavensis*, green; or *D. grimshawi*, red).

Once the initial *e*BLAT alignments are completed, the *EvoPrinterHD *intra-genomic comparative algorithm automatically determines: (1) the number of aligning bases in the second and third *e*BLAT alignments that are not identified in the first (highest scoring) alignment for each species, called the "R" value indicating putative rearrangements in the test species, (2) the number of aligning bases in the second and third alignments that are also aligning in the highest score alignment, termed the "D" value for putative duplications, and (3) the number of aligning bases that are shared by all three alignments, indicating conserved sequences within putative repetitive elements. For example, the alignment scorecard of a *D. melanogaster *3,570 bp input reference sequence, located 6 kb 5' to the *fushi tarazu *gene, reveals that 5 of the 11 species included in the analysis have undergone putative rearrangements in their aligning regions compared to the reference genome (Figure 4). The rearrangements within 4 of the 5 genomes (*D. mojavensis, D. grimshawi, D. willistoni *and *D. virilis*) flank the aligning bases in each of their highest score aligning regions (noted by the color coded number in the R column) (Figure 4). c*e*BLATs of these 5 species identified that each contained at least two different MCS rearrangements relative to the input *D. melanogaster *reference DNA (Figure 5A and data not shown).

### Generating EvoPrints, and EvoDifferences profiles and EvoUnique Prints

Based on the data provided on the alignment scorecard, different combinations of c*e*BLAT alignments can be chosen to generate an *EvoPrint*. The *EvoPrinter *algorithm [5] creates an array of nucleotide strings from each of the selected alignments and then looks for conservation of sequence by looping through each of the strings one base at a time, outputting an uppercase base for only those input reference DNA nucleotides that are aligned in all of the different c*e*BLATs included in the analysis (Figure 5B). Those DNA bases within the input DNA that are not shared with all species are represented as lowercase nucleotides. The "All Alignments or None" options for each species allows for rapid changes in the repertoire of species alignments used to generate an *EvoPrint*. As a default setting, *EvoPrinterHD *selects c*e*BLATs to generate an *EvoPrint*; however, the user can select just the highest scoring alignment to generate an *EvoPrint*, and doing so eliminates potential false positives that are identified as repeat sequences. As discussed above, when evolutionarily distant species are included in the analysis, MCS containing genomic rearrangements in one or more of the selected genomes are identified in the second and third *e*BLAT alignments. To include the rearranged sequences in the analysis, c*e*BLATs are used to generate the *EvoPrint*. The use of the intra-species c*e*BLATs in the *EvoPrint *procedure, rather than selecting first, second or third alignments for generation of the *EvoPrint*, enhances the ability of *EvoPrinterHD *to identify and display, in a single uninterrupted sequence, conserved sequences within the input DNA even though the MCSs reside within genomic rearrangements in one or more of the orthologous DNAs included in the comparative analysis. Our experience indicates that highly repetitious sequences do not interfere with the use of c*e*BLATs, because the presence and position of repeats varies across the species used to generate the *EvoPrint*. For the 20 vertebrate or for the enteric bacteria, genomes can be added or removed from the initial analysis simply by returning to the selection page and adding or deselecting different genomes. Because *EvoPrinterHD *holds the previous alignments in memory, the time required to add additional genomes to the comparative analysis is significantly reduced.

An additional readout, the *EvoDifferences *profile, is also displayed along with the *EvoPrint*; it highlights the unique differences (conserved sequence losses) that each species contributes to the comparative analysis (Figures 2B and 5C). The *EvoDifferences *profile can also be considered a "relaxed *EvoPrint*" since bases identified by the different colors are present in all species except for the single species denoted by that color. The apparent absence of a conserved sequence or base change in a single species could have several explanations: (1) the difference represents a unique evolutionary change, (2) it may be the result of a sequencing error, and/or (3) the sequence is present but not identified by the c*e*BLAT due to three or more genomic rearrangements in the aligning region.

For bacteria, a third readout, the color-coded *EvoUnique *print, highlights those bases in the input reference DNA that are unique (that do not align with any of the other genomes included in the analysis) and those bases that align with only a single other or two other genomes included in the analysis (data not shown).

### Parsing and curation of selected conserved sequences

To facilitate the comparative analysis of different conserved sequences from different enhancers, *EvoPrinterHD *allows for the curation of CSBs by enabling the user to automatically extract and collate CSBs in both forward and reverse-complimented orientations (data not shown). The "extract conserved sequence block" option (located at the top of each *EvoPrint *readout) provides for the automatic extraction, naming and consecutive numbering of 6 bp or longer CSBs from selected regions of an *EvoPrint *or *EvoDifferences *profile (see tutorial [9]). In addition to the annotated list of forward and reverse sequences the readout shows the selected *EvoPrinted *region from which the conserved sequences were extracted. A link is also provided to the *cis*-Decoder CSB comparative algorithms [4].

### Identifying species-specific changes in less-conserved DNA

**Figure 6:**
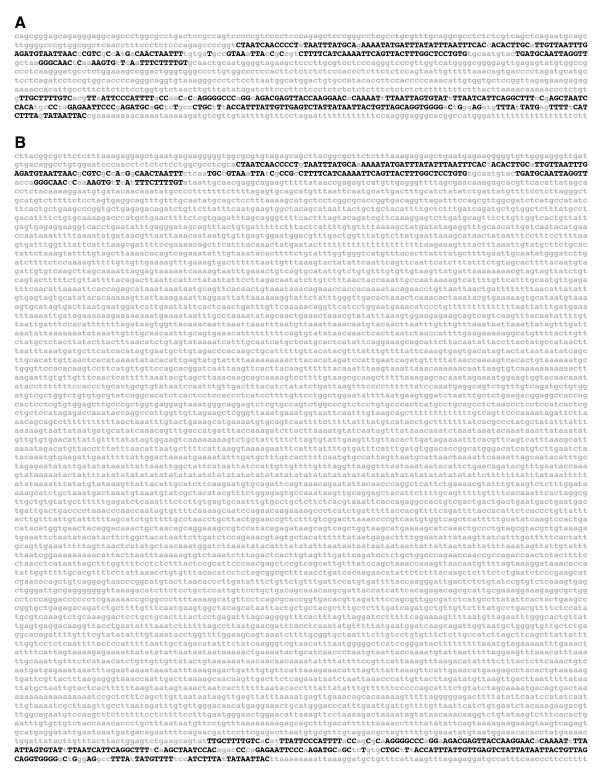
**Genome-specific flexibility in less-conserved sequences revealed by exchanging input reference DNAs**. By swapping the input reference DNA for one of the aligning regions in another genome and reinitiating the *EvoPrint *analysis, one can identify species-specific changes in the spacing between conserved sequences. A) *EvoPrint *analysis of the human CASZ1 gene identified two highly conserved MCSs within its second intron that are separated by 441 bp. Shown is a relaxed *EvoPrint *that was generated with c*e*BLAT alignments of the human sequence to: chimpanzee, rhesus, mouse, rat, dog, cat, horse, cow, hedgehog, elephant, armadillo, opossum, chicken, *X. tropicalis, Fugu, Tetraodon, Medaka*, stickleback, and zebrafish genomes. Uppercase black-colored bases are present in all orthologs or found in all but one of the aligning regions. B) Shown is a relaxed *EvoPrint *obtained when the human input reference sequence, used to generate the *EvoPrint *shown in (A), is exchanged for the highest scoring aligning region in the zebrafish genome. The zebrafish CASZ1 relaxed EvoPrint reveals that the intervening genomic region between the two highly conserved MCSs in the zebrafish orthologue is 7,061 bp longer than that found in the human genome.

*EvoPrinterHD *allows for the rapid exchange of the input reference DNA; it draws from memory the genomic sequence of the highest aligning region of any species identified in the initial analysis. Once a change in reference DNA is requested (at the additional alignment options page [8]), the alignment process is automatically reinitiated using the highest scoring aligning region of the selected genome as the new input reference DNA. Figure 5 highlights the genome-specific variability of less-conserved sequences between vertebrate MCS regions. Within the second intron of the human *CASZ1 *gene [22], a homolog of the *Drosophila castor *gene [23,24], two highly conserved MCSs were identified that are each present once in most, if not all, vertebrate genomes. Using the human *CASZ1 *2^nd ^intron as the input reference DNA and all 20 vertebrate genomes, a relaxed *EvoPrint *reveals that the intervening distance between the MCSs in the human genome is 441 bp (Figure 6A). By exchanging the human sequence with the highest scoring aligning region from the zebrafish genome and repeating the analysis, the separation between the conserved sequence clusters was found to be 7,502 bp (Figure 6B). Both human and zebrafish relaxed *EvoPrints *identified the same conserved bases in the two MSC clusters with few exceptions, and the spacing between conserved sequence blocks within the MCSs remained almost unchanged. Additional reference DNA swapping revealed that the non-or less-conserved intervening sequence between these MCSs is quite variable. For example, in fish the length varied between 1,609 to 7,502 bp and in frogs and chickens the distance was 1,610 and 408 bp, respectively (data not shown).

## Conclusion

*EvoPrinterHD *affords a rapid, convenient way to detect and curate DNA sequence conservation between related and evolutionarily distant animals. When multiple genomes are included in the analysis, the uninterrupted *EvoPrint *readout provides a species-centric view of conserved sequences that are required for gene function. *EvoPrinterHD *advances the *EvoPrint *method by providing an automated higher-definition view of sequence conservation from which the conserved sequence blocks can be rapidly curated for subsequent analysis. *EvoPrinterHD *also identifies genomic regions within one or more of the selected species that harbor rearrangements of the conserved DNA, and identifies unique or uniquely shared DNA sequences within bacterial genomes.

## Methods

### Genome sequence files and their assembly dates

The following genome sequence files were curated from the Genome Bioinformatics Group of University of California, Santa Cruz [25]: Human, March 2006 (hg18); Chimpanzee, March 2006 (panTro2); Rhesus, January 2006 (rheMac2); Rat, November 2004 (rn4); Mouse, February 2006 (mm8); Cat, March 2006 (felCat3); Dog, May 2005 (canFam2); Horse, January 2007 (equCab1); Cow, March 2005 (bosTau2); Opossum, January 2006 (monDom4); Chicken, May 2006 (galGal3); *Xenopus tropicalis*, August 2005 (xenTro2); Zebrafish, March 2006 (danRer4); *Tetraodon*, February 2004 (tetNig1); *Fugu*, October 2004 (fr2); Stickleback, February 2006 (gasAcu1); *Medaka*, April 2006 (oryLat1); *D. melanogaster*, April 2006 (dm3); *D. simulans*, April 2005 (droSim1); *D. sechellia*, October 2005 (droSec1); *D. yakuba*, November 2005 (droYak2); *D. erecta*, August 2005 (droEre1); *D. ananassae*, August 2005 (droAna2); *D. pseudoobscura*, November 2005 (dp3); *D. persimilis*, October 2005 (droPer1); *D. virilis*, August 2005 (droVir2); *D. mojavensis*, August 2005 (droMoj2); *D. grimshawi*, August 2005 (droGri1); *C. elegans*, January 2007 (ce4); *C. brenneri*, January 2007 (caePb1); *C. briggsae*, January 2007 (cb3); *C. remanei*, March 2006 (caeRem2); and *P. pacificus*, February 2007 (priPac1); The genome sequence files for the Elephant, June 2005; Hedgehog, June 2006 and Armadillo, June 2005 were downloaded from the Broad Institute [26].

The following bacteria genome sequence files were curated from the BacMap database of University of Alberta [27]: *Staphylococcus aureus *COL; *Staphylococcus aureus *MRSA252; *Staphylococcus aureus *MSSA476, *Staphylococcus aureus *Mu50; *Staphylococcus aureus *MW2; *Staphylococcus aureus *N315; *Staphylococcus aureus subsp. aureus *NCTC 8325; *Staphylococcus aureus *RF122; *Staphylococcus aureus subsp. aureus *USA300; *Staphylococcus epidermidis *ATCC 12228; *Staphylococcus epidermidis *RP62; *Staphylococcus haemolyticus *JCSC1435; *Escherichia coli *536; *Escherichia coli *APEC O1; *Escherichia coli *CFT073; *Escherichia coli *O157:H7 EDL933; *Escherichia coli *K12 MG1655; *Escherichia coli *W3110; *Escherichia coli *O157:H7 *Sakai*; *Klebsiella pneumoniae *MGH 78578; *Salmonella enterica *Choleraesuis SC-B67; *Salmonella enterica *Paratypi A ATCC 9150; *Salmonella typhimurium *LT2; *Salmonella enterica *CT18; *Salmonella enterica *Ty2; *Shigella boydii *Sb227; *Shigella dysenteriae *Sd197; *Shigella flexneri *2a 2457T; and *Shigella flexneri *301. The genome sequence files for *Staphylococcus aureus subsp. aureus *JH1, *Staphylococcus aureus subsp. aureus *JH9, *Staphylococcus aureus *Mu3, and *Staphylococcus aureus subsp. aureus str*. Newman were curated from the European Bioinformatics Institute of the European Molecular Biology Laboratory [28]. The genome sequence file for *Escherichia coli *UT189 was taken from Enteropathogen Resource Integration Center [29], and genome sequence data for *Salmonella bongori *was downloaded from the Sanger Institute Sequencing Centre [30].

The mosquito genome sequence files for *Aedes aegypti*, *Anopheles gambiae *and *Culex pipiens *were curated from the VectorBase database [31].

## Authors' contributions

ASY, YL and YF participated in the design and implementation of the algorithms. JR participated in the web page design and tutorial. TB and WFO conceived the study, participated in the design and coordination of the algorithms and prepared the manuscript. All authors have read and approved the final draft of the manuscript.
